# Nonequilibrium Steady States in Active Systems: A Helmholtz–Hodge Perspective

**DOI:** 10.3390/e27050525

**Published:** 2025-05-14

**Authors:** Horst-Holger Boltz, Thomas Ihle

**Affiliations:** Institute for Physics, University of Greifswald, 17489 Greifswald, Germany

**Keywords:** nonequilibrium steady states, Helmholtz–Hodge decomposition, Freidlin–Wentzell

## Abstract

We revisit the question of the existence of a potential function, the Cole–Hopf transform of the stationary measure, for nonequilibrium steady states, in particular those found in active matter systems. This has been the subject of ongoing research for more than fifty years, but continues to be relevant. In particular, we want to make a connection to some recent work on the theory of Helmholtz–Hodge decompositions and address the recently suggested notion of typical trajectories in such systems.

## 1. Introduction

A remarkable property of nonequilibrium statistical physics in general and active matter physics [1,2,3] is the existence of steady states [4,5,6,7,8,9,10,11,12,13,14,15,16,17,18,19,20,21,22,23,24,25], that is, the existence of a stationary probability distribution for stochastic dynamics, even though there is (in the case of active matter in particular) manifestly no notion of detailed balance [26,27] in the system: a system is active because stored energy is consumed on the smallest considered scale. To make this more specific, a flying bird metabolizes food to sustain its motion, but taking these details into account for a description of the collective motion of a flock of birds is neither feasible nor reasonable. Hence, flocking [28,29] is routinely discussed using models that are not built on the bedrock fundamentals of classical mechanics: reciprocity as well as conservation of energy, momentum and angular momentum. A similar tradeoff, leaving the confinement of the familiar structure of Hamiltonian (or at least detailed balance) dynamics for the possibility to capture the effective dynamics of a coarse-grained system, can be found in many applications, including biology, physics, sociology and complex systems in general [22,30,31,32].

The remarkable success of equilibrium statistical mechanics has quite naturally lead to many forays to extend its machinery from equilibrium systems (which feature detailed balance in the stationary states) to nonequilibrium systems. As discussed elsewhere, this is somewhat straightforward for approaches based on kinetic theory [33,34] because they usually contain very limited assumptions about the structure of the dynamical equations, but this comes with the drawback that the necessary closure of the BBGKY hierarchy is usually only possible in some parameter ranges. The arguably most famous results in this field, the Boltzmann distribution and the concept of (as well as specific expressions for) entropy, are not readily adaptable. While it would be very desirable to be able to directly state the stationary distribution, there is no general expression, but there has been an extensive amount of work to find potential functions (the logarithm of the stationary distribution) in nonequilibrium systems with special structures.

In this paper, we revisit, review and rephrase some older notions in this regard [4,5,6,7,8,9,10,11,35], with a particular emphasis on active systems (see also refs. [23,25] for approaches to nonequilibrium distributions in these systems), and address the recently (re-)introduced notion of deterministic *typical* trajectories [21,36,37,38,39]. Serendipitously, some progress has been made in understanding the structure necessary for such a distribution, an orthogonal Helmholtz–Hodge decomposition of the associated generalized dynamics, from a mathematical perspective [40,41].

The structure of this paper is as follows: We introduce the relevant terms and definitions in the upcoming section, with an emphasis on the structure of equations as they are encountered in typical active matter applications, i.e., starting from overdamped Langevin equations. An equivalent formulation of the statistics is given by the Fokker–Planck equation and will be discussed thereafter. We show that the orthogonal Helmholtz–Hodge decomposition is the relevant condition for easily findable Boltzmann distributions. Then, we present a deterministic version of the stochastic system that is, therefore, described by a Liouville equation, giving rise to the typical trajectories. Some final thoughts are given at the end.

## 2. The Langevin Equation: Terms and Definitions

Throughout this work, we consider autonomous dynamical systems of the following form:

(1a) $$\begin{array}{cc} \dot{x}\left(t\right) & =-\nabla V(x)+u(x)+\lambda r(x)+\xi \end{array}$$

with a Gaussian noise ξ characterized by

(1b) $$\begin{array}{cc} \langle \xi_{i}\left(t\right)\rangle & =0 \end{array}$$

(1c) $$\begin{array}{cc} \langle \xi_{i}\left(t\right)\xi_{j}\left(t^{\prime }\right)\rangle & =2D\delta (t-t^{\prime }) \end{array}$$

and a deterministic flow field F=−∇V+u+λr that we have separated into three parts. This is performed such that for λ=0, the system has a *strictly orthogonal Helmholtz–Hodge decomposition* [40] (${\,}^{⊥}$HHD), i.e., the flow field $F_{\mathrm{HHD}}=-\nabla V+u$ has two contributions: a gradient flow −∇V and an additional solenoidal flow u that is orthogonal to the gradient flow,

(1d) $$\begin{array}{cc} \nabla \cdot u & =0 \end{array}$$

(1e) $$\begin{array}{cc} (\nabla V)\cdot u & =0. \end{array}$$

These conditions have long been recognized as relevant for the existence of a directly accessible potential function [8,9,10,20]. We want to consider a somewhat broader class of systems close to one with ${\,}^{⊥}$HHDby adding a small (λ≪1) otherwise unrestricted perturbation r to it. The relevant criterion for the smallness of λ will be the existence of a meaningful expansion of the stationary measure in terms of λ. We note that in practical situations this representation is ambiguous and a suitable criterion (this is partially discussed below) would be necessary to find the best representation for which the λ term is the least perturbation.

Before addressing our interest in this particular class of systems, it is useful to restate some recent results, due to Suda [40,41], regarding deterministic dynamical systems and the existence of ${\,}^{⊥}$HHD. If F has a ${\,}^{⊥}$HHD with potential function *V* (system of Equation (1a,1b,1c,1d,1e) with D=λ=0), then the ω-limit set [42] (the set of points the system reaches for t→∞) is given by [40] ∇V=0. In the absence of noise, the additional “driving” term u in this sense does not change the qualitative behavior of the system at long times; it will be found at some point with ∇V=0. This does not allow for conclusions on, e.g., the relative occupancy of multiple valleys in a general, more complex energy landscape or any dynamical quantities, but is an important indicator that a field u that does allow for ${\,}^{⊥}$HHD is not all too different from a gradient flow system. This is something that we will formalize below for stochastic systems. While there are approaches to creating a general Helmholtz–Hodge decomposition [43], there is to our knowledge currently no algorithm for or general theorem on the existence of ${\,}^{⊥}$HHD in general dimensions. However, there are some results known [41] for special cases; for example, a linear flow field F=Ax with $A\in R^{n\times n}$ normal ($[A,A^{T}]=0$) has a ${\,}^{⊥}$HHD. In two dimensions, Suda [41] gives explicit conditions and constructions for linear and quadratic flows. Some results of Liverpool [37] are also applicable here as the case of ${\,}^{⊥}$HHD corresponds to the special case of C=0 in the nomenclature of Ref. [37]. We will address this later.

It is also worth noting that there is a general class of systems with ${\,}^{⊥}$HHD that are of the form $F_{\mathrm{HHD}}=-\nabla V+u$ with u=A∇V, with a constant anti-symmetric matrix A, i.e, $A_{ij}=-A_{ji}$. A more thorough discussion of the planar case can be found in Ref. [44]. This links to the stochastic differential equation (SDE) decomposition [45,46,47] that has been considered and ascribed to a different interpretation of stochastic dynamics that considers the zero-mass rather than the overdamped limit [48]. As has been noted before [40], these two decompositions have significant overlap and are identical for linear flow, but there are explicit examples of flows with ${\,}^{⊥}$HHD that are not of the SDE type. A similar decomposition has also been discussed in Ref. [49].

## 3. Fokker–Planck Equation

The Fokker–Planck equation for the probability density function corresponding to the Langevin Equation (1a,1b,1c,1d,1e) is obtain by means of standard stochastic calculus [50,51],

(2) $$\begin{array}{cc} \partial_{t}P\left(x,t\right) & =\sum_{i}\nabla_{i}\left(-F_{i}P+D\nabla_{i}P\right)\\ \; & =-\nabla \cdot (-\nabla V+u+\lambda r)P+D\Delta P. \end{array}$$

In particular, we are interested in stationary solutions $P_{\mathrm{stat}}$ with $\partial_{t}P_{\mathrm{stat}}=0$. The most well-known class of such stationary distributions are *Boltzmann distributions* $P\propto e^{-V/D}$, which solve the stationary Fokker–Planck equation for gradient flow, u+λr=0. Having an explicit construction for the stationary measure is a huge analytical asset that makes equilibrium systems more easily treatable than nonequilibrium or active systems. However, certain instances of the latter do allow for nonequilibrium steady states (NESSs) and many avenues into formulating effective potentials have been proposed. Here, we utilize the explicit form of Equation (1a,1b,1c,1d,1e) to extend the validity of the Boltzmann distribution to systems with ${\,}^{⊥}$HHD and consider corrections to it.

We consider the Cole–Hopf transform *H* of the stationary distribution, i.e., $P_{\mathrm{stat}}\propto e^{-H}$. This quantity *H* is also called the stochastic entropy of the system, Refs. [21,39]. Similarly to approaches going back to McLennan [7,20,24,52], we assume a perturbative form $H=h+\lambda g+O(\lambda^{2})$ with $\partial_{\lambda }h=\partial_{\lambda }g=0$. Other variational approaches have been discussed in the literature [53,54,55,56,57,58,59,60]. We determine *h* and *g* by solving the stationary version of Equation (2) order by order in λ. For the ${\,}^{⊥}$HHD case of $O(\lambda^{0})$, this gives

(3) $$\begin{array}{cc} 0 & =-\Delta V+\left(\nabla V\right)\cdot \nabla h-u\cdot \nabla h-D{\left(\nabla h\right)}^{2}+D\Delta h. \end{array}$$

which is solved by

(4) $$\begin{array}{cc} h & =\frac{V}{D} \end{array}$$

even in the presence of a non-vanishing u as we demand u·∇V=0. This is the thermodynamic equivalent of the notion for deterministic (D=0) systems [40] that the ${\,}^{⊥}$HHD-compliant field u does not constitute a qualitative perturbation. In fact, the stationary measures for $F_{\mathrm{grad}}=-\nabla V$ and $F_{{\,}^{⊥}\mathrm{HHD}}=-\nabla V+u$ are exactly identical, which is perhaps not all too surprising on an intuitive level as u only evolves the system along lines of equal energy. While detailed balance is broken, these systems are *statistically indistinguishable* from equilibrium systems. That the stationary measure in a noisy gradient flow system can be sampled by means of an appropriately chosen non-reversible (active) flow is the rationale underlying non-reversible Monte Carlo techniques, see for example refs. [61,62,63]. An example of such a system in two dimensions is the noisy Hopf oscillator,

(5a) $$\begin{array}{c} \dot{x}=Ax-Bx^{2}x+\Omega x+\xi \end{array}$$

with x=|x| (the ${\,}^{⊥}$HHD conditions can be rephrased as $\left\{\Omega ,\Omega^{T}\right\}=\mathrm{Tr}\left(\Omega \right)=0$ in this case. In two dimensions, the given parametrization is thus unique) and

(5b) $$\begin{array}{cc} \Omega & =\left(\begin{array}{cc} 0 & \Omega \\ -\Omega & 0 \end{array}\right), \end{array}$$

considered in Ref. [37]. This system has a ${\,}^{⊥}$HHD with $V=-\frac{A}{2}x^{2}+\frac{B}{4}x^{4}$ and u=Ωx, as the gradient flow −∇V is always perpendicular to the solenoidal rotational flow u. Therefore, the stationary measure is indeed given by the Boltzmann distribution $P\propto e^{-V/D}$. In this case, there is an obvious interpretation of the ${\,}^{⊥}$HHD and the equivalency of the stationary measures: going into a co-rotating frame, we can eliminate Ω from the equations of motion and a rotationally invariant gradient flow remains. So in this case, the existence of the decomposition is a direct consequence of the symmetry of the system. Dynamically, the existence of u will of course change the behavior significantly as it adds a persistent circular motion onto the gradient descent.

A less trivial example considered by Suda [41] is

(6a) $$\begin{array}{cc} F & =\left(\begin{array}{c} x^{2}-2xy+3y^{2}\\ 4x^{2}-4xy+2y^{2} \end{array}\right) \end{array}$$

(6b) $$\begin{array}{cc} -\nabla V & =\frac{1}{26}\left(\begin{array}{c} -19x^{2}+18xy-7y^{2}\\ 9x^{2}+17y^{2}-14xy \end{array}\right) \end{array}$$

(6c) $$\begin{array}{cc} u & =\frac{1}{26}\left(\begin{array}{c} 45x^{2}-70xy+85y^{2}\\ 95x^{2}-90xy+35y^{2} \end{array}\right) \end{array}$$

for which it is straightforward to check that ∇·u=u·∇V=0.

Going back to the perturbative solution of the Fokker–Planck Equation (2), we now consider the order O(λ), which will give the first corrections to the Boltzmann distribution. We find

(7) $$\begin{array}{cc} 0 & =\nabla \cdot r-r\cdot \nabla h-(-\nabla V+u)\cdot \nabla g+D[-\Delta g-2(\nabla h)\cdot \nabla g]\\ \; & =\nabla \cdot r+D^{-1}r\cdot \nabla V+\left(\nabla V-u\right)\cdot \nabla g-D\Delta g-2\left(\nabla V\right)\cdot \left(\nabla g\right) \end{array}$$

where we used the zeroth order result h=V/D. Physically, any sufficiently large noise strength *D* will lead to a uniform distribution and the more relevant case is that of small noise. We thus write

(8) $$\begin{array}{c} g=\frac{\tilde{g}}{D}+O\left(D^{0}\right) \end{array}$$

and find that $\tilde{g}$ is the solution to

(9) $$\begin{array}{c} \left(\nabla V+u\right)\cdot \nabla \tilde{g}=r\cdot \nabla V. \end{array}$$

As this only fixes a directional derivative of $\tilde{g}$, we solve it (or give a formal representation) by considering the dynamical system

(10a) $$\begin{array}{cc} \dot{y} & =\nabla V+u. \end{array}$$

Note that this is not the same as the deterministic case of Equation (1a,1b,1c,1d,1e) with λ=0, as the sign of the gradient flow is different. We can determine the total change in $\tilde{g}$ along solutions of Equation (10a),

(10b) $$\begin{array}{cc} \frac{d}{dt}\tilde{g}\left(y\left(t\right)\right) & =\partial_{t}\tilde{g}+\dot{y}\cdot \nabla \tilde{g}=+r\cdot \nabla V \end{array}$$

which directly implies

(10c) $$\begin{array}{cc} \tilde{g}\left(y\left(t\right)\right) & =-\int_{y_{0}}^{y(t)}dt^{\prime }\;r\cdot \nabla V\equiv W_{r}. \end{array}$$

Thus, we have (at least formally) found that for λ,D≪1 the stationary solution to the Fokker–Planck Equation (2) is given by

(11) $$\begin{array}{cc} P_{\mathrm{stat}} & \propto e^{-(V+\lambda W_{r})/D} \end{array}$$

which is the appropriate generalization of the Boltzmann distribution.

As an example, we consider a simplified version of the noisy Hopf oscillator [37] of Equation (5a,5b) in two dimensions, $x={\left(x,y\right)}^{T}$, with a “relevant, active” perturbation $r=ye_{x}$. We set A=−1 and B=0, which eliminates the “Hopf oscillator” nature, but here we are only interested in an easily tractable example. We know that $h=x^{2}/\left(2D\right)$. We find (we note this solution is not perturbative in *D*, but only in λ, as the Laplacian does not contribute) $g=\left(xy-\Omega \left(y^{2}-x^{2}\right)\right)/\left(2D^{*}\right)$, with an effective noise $D^{*}=D\left(1+\Omega^{2}\;/\;2\right)$. This is somewhat similar to the concept of effective temperatures in out-of-equilibrium systems [64,65]. Thus, a solenoidal flow field that does not change the statistics in a system with ${\,}^{⊥}$HHDwill effectively serve as an additional source of noise once that property is broken by a small perturbation. Intuitively, this is expected to be a generic effect.

Interestingly, one can also approach the full problem, e.g., for A=B=1, with some work and assuming a perturbative structure in Ω. This leads to $g=\left(xy-\Omega \left(y^{2}-x^{2}\right)\log\left[{\left(1-\left(x^{2}+y^{2}\right)\right)}^{2}\right]/\left(4\left(x^{2}+y^{2}\right)\right)\right)/\left(2D^{*}\right)$. The logarithmic contributions (and, particularly, the singularity in *g* for $x^{2}+y^{2}\to 1$) indicate that perturbative approaches are of limited use, as the proper solution will in general [66,67] not be analytic (with respect to both the coordinates and the parameters).

Having established that these dynamical systems, which allow for a ${\,}^{⊥}$HHD feature equilibrium Boltzmann distributions and how to correct this for weakly perturbed systems, we ultimately progress to the concept of *typical trajectories* proposed in Ref. [37] as a means of studying nonequilibrium steady states, but first, an aside on systems with relevant inertia.

### Inertial Systems

In classical mechanics, systems such as Equation (1a,1b,1c,1d,1e) occur as the overdamped limit of the equation of motions with a dissipative force and noise in form of thermal forces, and one considers

(12) $$\begin{array}{cc} \left(\begin{array}{c} \dot{x}\\ \dot{p} \end{array}\right) & =J\nabla H-\gamma \left(\begin{array}{c} 0\\ p \end{array}\right)+\left(\begin{array}{c} 0\\ \xi \end{array}\right) \end{array}$$

with a Hamiltonian H and the symplectic matrix

(13) $$\begin{array}{cc} J & =\left(\begin{array}{cc} 0 & 1\\ -1 & 0 \end{array}\right). \end{array}$$

It is straightforward to see that the statements made before still hold: the Boltzmann distribution does give the corresponding stationary measure and this remains true if an additional solenoidal flow u is included in Equation (12) as long it is strictly orthogonal to the gradient flow ∇H. A further perturbation λr, which does not allow for such a decomposition by means of an effective new Hamiltonian and orthogonal solenoidal flow field, will lead to a change in the Cole–Hopf transform that is determined by an equation that is qualitatively equivalent to Equation (7). Thus, we opt to not further consider inertia here as the different geometry does not induce a significant change.

## 4. Mapping to a Liouville Equation: Typical Trajectories

Using the notation of the earlier section for the Cole–Hopf transform of the stationary distribution, $H=-\log P_{\mathrm{stat}}$, but not making any assumptions about the flow field F, a class of trajectories has attracted interest recently [37,38] that we will name typical trajectories, following Ref. [37]. These are solutions given by the “mean velocity”, a concept that has long been considered, see for example Ref. [36] (which calls the additional term, D∇H, the osmotic velocity) or Ref. [21], and, therefore, subject to the deterministic dynamics given by

(14) $$\begin{array}{cc} \dot{X}\left(t\right) & =F+D\nabla H. \end{array}$$

By construction, they have the property that the stationary solutions to the *Fokker–Planck* Equation (2) also solve the *Liouville* equation for this deterministic system. This mapping of stochasticity onto deterministic interaction (with the stationary probability effectively being an external field) has been identified as favorable for some inference applications, as the probability distribution can be easily approximated empirically [38]. However, determining *h* analytically is a severe technical challenge in general systems. One possible application of these trajectories is that they offer a way to establish some insight into the convergence to the attractor that is represented by *h* [37,68].

Additionally, the proposed “typical” trajectories do convey some information in the limit of weak noise, as they locally represent the trajectory between points that maximizes the transition probability among all trajectories in the weak-noise approximation. However, phase space (de-)compression is possible and arguably typical in active matter systems with generalized interactions, with one consequence being that, in general, *Theorem 1a* of Ref. [37] (depending on the reader’s understanding) either does not hold (the body derivative of the probability distribution along the trajectories does not vanish) or is trivial (the stationary distribution is stationary). In fact, in most non-trivial systems there will be no meaningful orbits along which the steady-state probability is constant, potentially limiting the usefulness of the proposed concept of *generalized steady states* [37]. In the following, we will elaborate on this point. The main point is that the alluring reduction of these nonequilibrium steady states to a single deterministic Ersatz system comes with a severe drawback: this system’s claim to convey representative information becomes arbitrarily meaningless. Thus, employing such notions to build upon on them as useful tools is very limited in their applicability [69]. One possible class of systems that are effectively close to ${\,}^{⊥}$HHD that is very pertinent to active matter in particular is systems displaying *motility-induced phase separation* or MIPS [70]. These systems of self-propelled particles with sterical interactions separate into essentially two types of motility: some particles are arresting each other and therefore are not moving (giving rise to trivial dynamics) while the other particles are mostly freely moving (which is solenoidal). The actual stationary measure is flat for symmetry reasons, but if this is broken by, e.g., pinned particles (similar to introducing an infinitesimal field in the Ising model to break the symmetry), ideas along these lines can still have merit.

In general, these solutions support a very liminal claim to typicality, as can be seen by the evolution of the probability itself. Its total change along a typical trajectory is given by

(15) $$\begin{array}{cc} \frac{dP}{dt}\left(X,t\right) & =\dot{X}\cdot \nabla P+\partial_{t}P. \end{array}$$

For the stationary distribution ρ(X), Equation (15) gives

(16) $$\begin{array}{cc} \frac{d\rho }{dt}\left(X\right) & =\dot{X}\cdot \nabla \rho =\left(F+D\nabla H\right)\cdot \left(-\nabla H\right)\rho \\ \; & \overset{\left(3\right)}{=}-\rho \sum_{i}\left(D\nabla_{i}^{2}H+\nabla_{i}F_{i}\right). \end{array}$$

For an F with ${\,}^{⊥}$HHD, the steady-state solution is given by Boltzmann weights h=V/D and the terms in (16) do indeed cancel out, corresponding to C=0 in the notation of Ref. [37]. For clarity, we note that this is analogous to but not identical with Liouville’s theorem from textbook mechanics as (1a,1b,1c,1d,1e) is not a Hamiltonian flow, in the absence of a symplectic structure. For truly active systems with general interactions, however, there is generally no analogon to Liouville’s theorem: the flow in phase space is compressible. In the language of kinetic theory, this amounts to a change in the superposition principle [71] by a phase compression factor, see for example Refs. [72,73] for a specific discussion of a model of active matter with orientational interactions, which constitutes a highly relevant model class of prototypical models [29] with generalized, active dynamics (which also can be explicitly non-reciprocal).

To put this point in a different, equivalent language, the stochastic entropy production [21,39,74,75], that is, the rate of change of the Cole–Hopf transform, is trivial for systems with ${\,}^{⊥}$HHD. Plugging the Liouville equation $\partial_{t}P=-\nabla \cdot \left(\dot{X}P\right)$ into Equation (15), we find

(17) $$\begin{array}{cc} \frac{dP}{Pdt}=\dot{H} & =-\nabla \cdot X=-\lambda \nabla \cdot r. \end{array}$$

Thus, there is *locally* no stochastic entropy production in systems with ${\,}^{⊥}$HHD. One could say they *typically* display detailed balance. Stationarity, however, only requires global balance, that is, no entropy production over the full orbit. As we can see, this is the general case for systems with finite λ, i.e., systems that cannot be decomposed in this way.

As an interesting aside, we note that the deterministic limit of Equation (16) not only implies a direct expression for the stationary distribution, $\rho \propto e^{-\int \;d\;t\nabla_{i}F_{i}}$, but by comparison to the propagator (or the Onsager–Machlup action therein) found for example by path integration methods [76,77] is a direct hallmark of the often overlooked notion that Langevin systems of this kind are most naturally considered in the Hänggi–Klimontovich (or “kinetic”) interpretation [47,78,79], which comes with the drawback of being non-causal at times.

We make the drawbacks implied by Equation (16) explicit for the case of the Brusselator, as considered in Ref. [37]. In this two-dimensional system, the dynamics of $x={\left(x,y\right)}^{T}$ are given by

(18a) $$\begin{array}{cc} \dot{x} & =F+\xi \end{array}$$

(18b) $$\begin{array}{cc} F & ={\left(\mu +x^{2}y-\left(\lambda +1\right)x,\lambda x-x^{2}y\right)}^{T}. \end{array}$$

**Figure 1:**
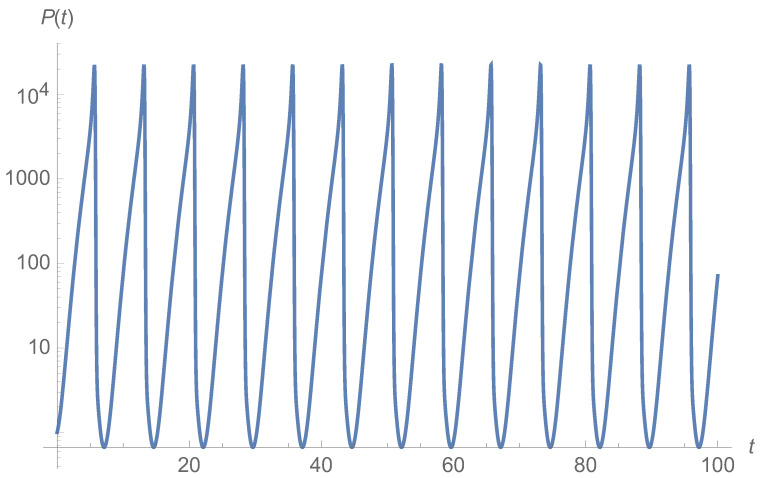
Time evolution of $P\propto e^{-h(X(t))}$, highlighting that the probability is not conserved along the trajectory, but varies. Here, we use the approximate expansion solution from Ref. [37], the ratio to a numerically found empirical distribution (cp. Figure 2), which varies by orders of magnitudes along the typical trajectory. This is partially a shortcoming of the expansion order, but the actual distribution is also not constant along any meaningful trajectory.

**Figure 2:**
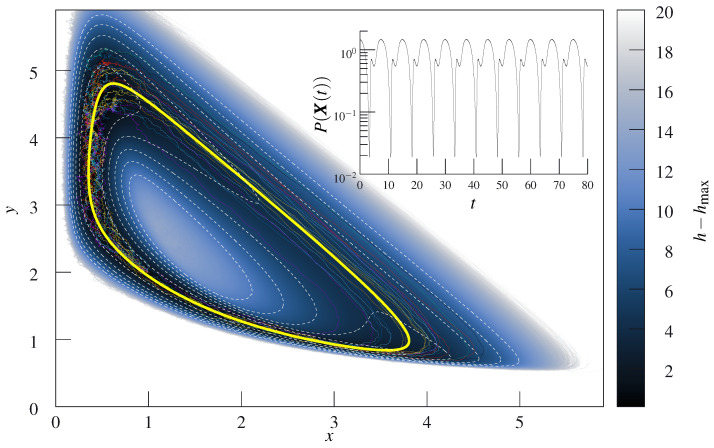
Cole–Hopf transform *h* of the empirically found stationary measure, $P_{\mathrm{steady}}\propto e^{-h}$, for the Brusselator system described in the main text. We added (dashed, white) equipotential lines as visual aids. The image visually corroborates the finding of the text and Figure 2 that in this system there are no orbits around the high-energy peak in the middle along which $P_{\mathrm{steady}}$ is constant that could be considered typical. The suggested typical trajectory of Liverpool is shown in yellow, and some exemplary trajectory as dotted lines around it. The bundle of trajectories is tightly focused on parts of the orbit and rather dispersed on others. The distribution was found from direct integration of the equations of motion (18b) and comprises roughly $10^{13}$ time steps in the steady state. We highlight the fact that *P* is not constant along the suggested X trajectory in the inset (scaling such that the shown quantity is effectively *h*). The relative spread in the steady-state probability along the suggested trajectory (14) is around two orders of magnitude in this system. From the picture alone, it is also evident that this would still occur if the orbit followed −∇h and, thus, was located directly in the canyon of the *h*-landscape.

**Figure 3:**
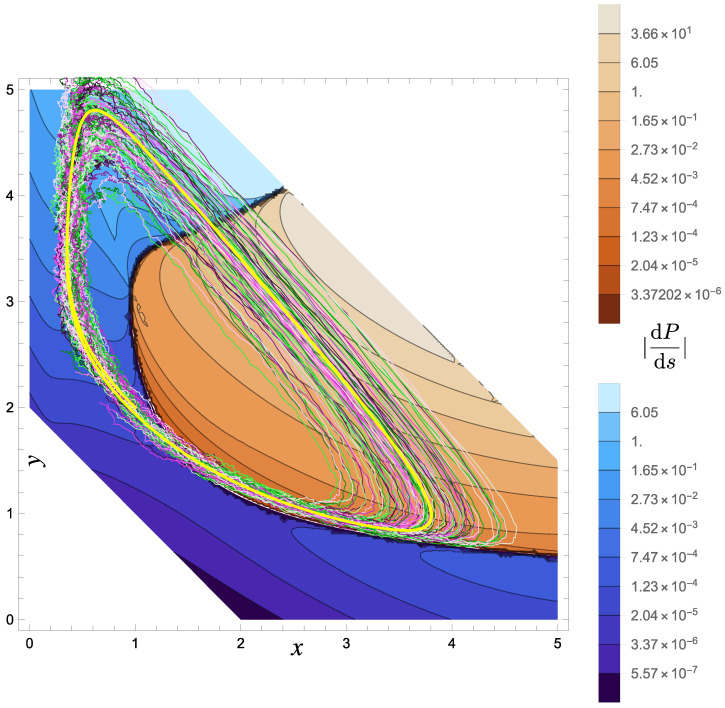
Color map of the change in probability along a trajectory, $\frac{dP}{ds}=\frac{dP}{dt}/\left|\dot{x}\right|$, based on the approximate stationary solution for the Brusselator with μ=1, λ=3 and $D=1/2{\left(0.1\right)}^{2}$ given in Ref. [37]. The color coding is conducted on a logarithmic scale, with reddish colors corresponding to negative values and blueish values to positive values. The thick yellow line corresponds to the typical trajectory X. The discussed effect is also apparent by considering an ensemble (here N=100) of trajectories (colored in green to white to pink, for visual clarity). The bundle of trajectories is tightly focused on parts of the orbit and rather dispersed on others. Thus, we argue that any sense of typicality is very limited to the parts of the orbit along which there is no phase space compression.

One possible, non-orthogonal Helmholtz–Hodge decomposition could be $H_{\mathrm{sol}}=-x^{3}y/3+x^{4}/12+\left(\lambda +1\right)/2\;x^{2}$ and $w_{\mathrm{sol}}={\left(\mu +x^{3}/3,\lambda x-x^{3}/3-x^{2}y\right)}^{T}$ and there exists no ${\,}^{⊥}$HHD with polynomial flow fields. Interestingly, there is a ${\,}^{⊥}$HHD for the linearized flow around the fixed point for essentially any parameters [41]. As such, this system—as long as it close to the fixed point, which is commonly assumed anyhow—is a good candidate for a system with a useful nonequilibrium steady state that is close to ${\,}^{⊥}$HHD decompositionability. For a system with μ=1, λ=3 and $D={\left(0.1\right)}^{2}/2$, an approximate solution for *h* is given in Ref. [37]. We have checked that this solution is indeed a solution to the stationary Fokker–Planck equation to the stated order (albeit neither stable nor accurate for large arguments, which is irrelevant here). As we show in Figure 1, the typical trajectories found from integrating Equation (14) for this stationary distribution do not remotely correspond to constant values of *h*. We present *h* as inferred from direct numerical integration of the stochastic equations of motion, Equation (18b), in Figure 2. We illustrate this further by depicting the change in the value of the stationary distribution along a trajectory for both the deterministic typical trajectory X as well as an ensemble of stochastic trajectories, see Figure 3.

However, the notion of typicality attributed to solutions of Equation (14) is warranted if the system can be meaningfully expanded around vanishing noise, D→0. In this *weak noise* case, the stationary distribution can be written as $P_{\mathrm{steady}}\propto e^{-g/D}$ with $g=H_{0}+DH_{1}+O\left(D^{2}\right)$. Using this Ansatz and separating terms by orders of *D*, the Fokker–Planck equation can be reduced to an effective Hamilton–Jacobi equation for $H_{0}$ and the so-called transport equation for $H_{1}$, see for example Refs. [80,81] for instructive discussions and Ref. [82] for a recent (physics) review. Their respective time evolutions are given by

(19) $$\begin{array}{cc} \partial_{t}H_{0} & =-F\cdot \nabla H_{0}-{\left(\nabla H_{0}\right)}^{2} \end{array}$$

(20) $$\begin{array}{cc} \partial_{t}H_{1} & =-\nabla \cdot F-2\left(\nabla H_{0}\right)\cdot \left(\nabla H_{1}\right)-F\cdot \nabla H_{1}+\Delta H_{0} \end{array}$$

The former equation is the Hamilton–Jacobi equation for a Hamiltonian system with the so-called Freidlin–Wentzell Hamilton [83] function $H_{\mathrm{FW}}=p^{2}+F\cdot p$ wherein the canonical moments are given by $p_{i}=\frac{1}{2}\left({\dot{x}}_{i}-F_{i}\right)$. The subspace of interest is p=0. As pointed out before [80], the solution to the latter equation, the transport equation, ultimately gives rise to a weak-noise propagator whose amplitude is modulated by

(21) $$\begin{array}{cc} A & \propto e^{-\int_{0}^{t}\mathrm{Tr}\left(\frac{\partial^{2}H_{\mathrm{FW}}}{\partial x\partial p}\left(t^{\prime }\right)\right)dt^{\prime }}. \end{array}$$

In the relevant subspace, the integrand becomes

(22) $$\begin{array}{c} {\left.\mathrm{Tr}(\frac{\partial^{2}H_{\mathrm{FW}}}{\partial x\partial p})\right|}_{p=0}=\nabla \cdot F \end{array}$$

rephrasing the point that the divergence of the generalized forces corresponds to phase space compression. Going back to the approach of Ref. [37], we can consider trajectories $X=X_{0}+DX_{1}+O\left(D^{2}\right)$. From the stationary versions of Equations (19) and (15), we directly find that

(23) $$\begin{array}{cc} 0 & ={\dot{X}}_{0}-F-\nabla H_{0} \end{array}$$

(24) $$\begin{array}{cc} 0 & ={\dot{X}}_{0}\nabla H_{1}+{\dot{X}}_{1}. \end{array}$$

Thus, the typical trajectories [37] are the zeroth order contribution. Before contemplating this further, it is interesting to explicitly state the next order. Using (20), we find

(25) $$\begin{array}{cc} 0 & =\nabla \cdot F+\left({\dot{X}}_{1}-\nabla H_{1}\right)\nabla H_{0}+\Delta H_{0}; \end{array}$$

one reasonable solution is ${\dot{X}}_{1}=\nabla H_{1}+q\nabla H_{0}$ with

(26) $$\begin{array}{c} q=-\frac{\nabla \cdot F+\Delta H_{0}}{{\left(\nabla H_{0}\right)}^{2}}. \end{array}$$

This is far from unique, as locally any vector field orthogonal to $\nabla H_{0}$ could be added. In a general setting, the limit D→0 is peculiar with respect to stationary distributions. In a chaotic system with a non-hyperbolic attractor (which is the generic case for an interacting system with a useful stationary state), the existence of a stationary probability (which does not depend on the initial state) is directly bound to the finiteness of the noise, i.e., D>0 [84].

Additionally, and more in line with the reasoning earlier, the typical trajectories are also trajectories along which the stationary distribution is close to constant, if the weak-perturbation analysis of the previous section is meaningful and the system is in this sense close to having a ${\,}^{⊥}$HHD. In this case, the typical trajectory is a solution of

(27) $$\begin{array}{cc} \dot{X} & =(-\nabla V+u+\lambda r)+D\nabla (h+\lambda g)\\ \; & =u+\lambda r+\lambda \nabla W_{r}. \end{array}$$

The total change in the the stationary distribution $P_{\mathrm{stat}}$ along these trajectories is of order O(λ),

(28) $$\begin{array}{cc} \frac{d}{dt}P_{\mathrm{stat}}\left(X\right) & =\left(u+\lambda r+\lambda \nabla g\right)\left(-\nabla h-\lambda \nabla g\right)P_{\mathrm{stat}}\\ \; & =\frac{\lambda }{D}\left(-\left(\nabla V+u\right)\cdot \nabla W_{r}-r\cdot \nabla V\right)P_{\mathrm{stat}}\\ \; & =-\frac{2\lambda }{D}r\cdot \nabla V. \end{array}$$

Here, we made use of Equation (9). This expression might serve as good test for the applicability of these typical trajectories, as λ, the distance to ${\,}^{⊥}$HHD, explicitly quantifies the amount “non-trival” activity in the system. However, it comes with the drawback that the specific construction is dependent on the choice of the underlying ${\,}^{⊥}$HHDdecomposition. Thus, it implies an optimal (for this discussion) choice that minimizes the overall change along the typical trajectory and gives a unique assessment of the distance to equilibrium [74]. Similarly, one could use the average active potential in the system as a gauge to this end. This will be explored in future work.

Finally, typicality is also easily established for systems, in which the steady state is such that all the detailed balance breaking fluxes in the nonequilibrium steady state (*NESS*) are *locally* trivial. One class of such systems is composed of systems that have global flocking states. There are two types of generalized forces here that could give rise to change along the typical trajectory: the propulsion and the alignment interactions. In a flocking state, the alignment interaction can effectively be of a gradient-type in a co-moving frame as changes in neighborhood topology can be negligible. If the propulsion speed then does not vary spatially or based on orientation (which it can [85]), the intricacies of activity outlined here vanish.

## 5. Discussion

We have (re)-introduced a perspective onto stationary probability distributions in nonequilibrium steady states (NESSs) that is rooted in a particular case of the Helmholtz–Hodge decomposition in which the deterministic part of the dynamical flow is made up of a gradient flow and a solenoidal flow that are orthogonal to each other. While this is functionally a rather old idea, some of the connections have been missing in the recent discussion in the literature. We argue that while such systems in general do not feature detailed balance they are trivially active as the stationary picture is identical to the equilibrium (pure gradient flow) case. In particular, we very explicitly show that the concept of typical trajectories, which has been proposed to shed light on nonequilibrium orbits, is strictly only justifiable for systems whose structure is very close to one with an orthogonal Helmholtz decomposition. For general systems (even already in the example system considered here, the planar Brusselator), the body derivative of the probability distribution along these typical trajectories will vary drastically, which can be related back to the idea of phase space compression. This is a hallmark of generalized dynamics whereas it is impossible in Hamiltonian mechanics as per Liouville’s theorem.

We relate this to the production of stochastic entropy with the result that there is no entropy production along the typical trajectories as long as the system can be decomposed in the proposed manner. As such, the decomposition can be phrased as a more relaxed notion of detailed balance: the stationary distribution is equivalent to the equilibrium potential of the related gradient flow as long as the system *typically* (in the sense of along typical trajectories) displays detailed balance. Detailed balance, whether strictly or typically, is, however, only a sufficient condition for stationarity and should not be assumed in general.

For a slightly more general class of systems close to an orthogonal Helmholtz–Hodge decomposition, we also discuss how to adapt the classical Freidlin–Wentzell expansion around deterministic systems.

We end with two conclusions. For one, the intricacies in finding suitable Boltzmann distributions (or potential functions) in systems with generalized dynamics highlights the relevance of also exploring the complementary approach of kinetic theory [33,34] in active matter and nonequilibrium physics: systematic derivations of approximate meso- and macroscalic descriptions starting from the microscopic equations of motion. These approaches are fundamentally less reliant on assumptions about the structure of the dynamical system. Secondly, we think more constructive approaches to explicitly find optimal (minimal λ) *orthogonal* Helmholtz–Hodge decompositions (extending the unconstrained tool-set laid out in works such as Ref. [43]) would greatly benefit future work and, thus, have to be considered a worthwhile direction of future (mathematical) research.

## Data Availability

Data and source code available upon request from the authors.
